# Mutant Mice With Calcium-Sensing Receptor Activation Have Hyperglycemia That Is Rectified by Calcilytic Therapy

**DOI:** 10.1210/en.2017-00111

**Published:** 2017-06-02

**Authors:** Valerie N. Babinsky, Fadil M. Hannan, Reshma D. Ramracheya, Quan Zhang, M. Andrew Nesbit, Alison Hugill, Liz Bentley, Tertius A. Hough, Elizabeth Joynson, Michelle Stewart, Abhishek Aggarwal, Maximilian Prinz-Wohlgenannt, Caroline M. Gorvin, Enikö Kallay, Sara Wells, Roger D. Cox, Duncan Richards, Patrik Rorsman, Rajesh V. Thakker

**Affiliations:** 1 Radcliffe Department of Medicine, Oxford Centre for Diabetes, Endocrinology and Metabolism, University of Oxford, Oxford OX3 7LE, United Kingdom; 2 Department of Musculoskeletal Biology, Institute of Ageing and Chronic Disease, University of Liverpool, Liverpool L7 8TX, United Kingdom; 3 Biomedical Sciences Research Institute, Ulster University, Coleraine BT52 1SA, United Kingdom; 4 Medical Research Council Mammalian Genetics Unit and Mary Lyon Centre, Medical Research Council Harwell Institute, Harwell Science and Innovation Campus, Oxfordshire OX11 0RD, United Kingdom; 5 Department of Pathophysiology and Allergy Research, Medical University of Vienna, Vienna A-1090, Austria; 6 GlaxoSmithKline Clinical Unit, Cambridge CB2 0GG, United Kingdom

## Abstract

The calcium-sensing receptor (CaSR) is a family C G-protein–coupled receptor that plays a pivotal role in extracellular calcium homeostasis. The CaSR is also highly expressed in pancreatic islet *α*- and *β*-cells that secrete glucagon and insulin, respectively. To determine whether the CaSR may influence systemic glucose homeostasis, we characterized a mouse model with a germline gain-of-function CaSR mutation, Leu723Gln, referred to as *Nuclear flecks* (*Nuf)*. Heterozygous- (*Casr^Nuf/+^*) and homozygous-affected (*Casr^Nuf/Nuf^*) mice were shown to have hypocalcemia in association with impaired glucose tolerance and insulin secretion. Oral administration of a CaSR antagonist compound, known as a calcilytic, rectified the glucose intolerance and hypoinsulinemia of *Casr^Nuf/+^* mice and ameliorated glucose intolerance in *Casr^Nuf/Nuf^* mice. *Ex vivo* studies showed *Casr^Nuf/+^* and *Casr^Nuf/Nuf^* mice to have reduced pancreatic islet mass and *β*-cell proliferation. Electrophysiological analysis of isolated *Casr^Nuf/Nuf^* islets showed CaSR activation to increase the basal electrical activity of *β*-cells independently of effects on the activity of the adenosine triphosphate (ATP)–sensitive K^+^ (K_ATP_) channel. *Casr^Nuf/Nuf^* mice also had impaired glucose-mediated suppression of glucagon secretion, which was associated with increased numbers of *α*-cells and a higher *α*-cell proliferation rate. Moreover, *Casr^Nuf/Nuf^* islet electrophysiology demonstrated an impairment of *α*-cell membrane depolarization in association with attenuated *α*-cell basal K_ATP_ channel activity. These studies indicate that the CaSR activation impairs glucose tolerance by a combination of *α*- and *β*-cell defects and also influences pancreatic islet mass. Moreover, our findings highlight a potential application of targeted CaSR compounds for modulating glucose metabolism.

## Teaser

Mice with a germline gain-of-function CaSR mutation have hypoinsulinemia, hyperglucagonemia, reduced pancreatic islet mass, and impaired glucose tolerance, which is rectifiable by calcilytic therapy.

Glucose homeostasis is tightly regulated by the joint actions of insulin and glucagon, which are secreted from the pancreatic islet *β*- and *α*-cells, respectively (1, 2). Diabetes mellitus is a bihormonal disorder that affects >330 million people worldwide and is characterized by reduced insulin secretion and aberrant glucagon secretion, which arises from alterations in islet function as well as mass (1, 2). G-protein–coupled receptors (GPCRs), which comprise the largest superfamily within the human proteome and are targeted by 40% of all currently approved drugs (3), facilitate the effects of diverse extracellular stimuli, ranging from fatty acids to neurotransmitters and gut hormones, on *α*- and *β*-cells and represent an exploitable target for the modulation of glucose homeostasis (4, 5). The extracellular calcium (Ca^2+^_o_)-sensing receptor (CaSR) is a family C GPCR that plays a key role in the parathyroid and renal regulation of Ca^2+^_o_ homeostasis by coupling to intracellular signal transduction cascades that include the G_q/11_-mediated stimulation of phospholipase C, which increases inositol 1,4,5-trisphosphate, thereby leading to a rapid rise in cytosolic calcium (Ca^2+^_i_) concentrations, and activating the mitogen-activated protein kinase pathway (6). The CaSR is also highly expressed in pancreatic islet *α*- and *β*-cells (7, 8), and studies involving isolated human islets and insulin-secreting cell lines have shown that activation of the CaSR following exposure to elevated Ca^2+^_o_ concentrations or allosteric activators triggers transient stimulations of insulin and glucagon secretion, which were associated with upregulation of phospholipase C and mitogen-activated protein kinase–mediated signaling responses (8–10). Moreover, a study involving wild-type (WT) mice has demonstrated pancreatic islet CaSR expression to be associated with insulin secretion *in vivo* (11). However, the role of this GPCR in systemic glucose homeostasis is unclear. For example, one patient-based association study has reported a common coding region CaSR gene variant to be an independent determinant of plasma glucose concentrations (12), whereas another study of patients with familial hypocalciuric hypercalcemia (FHH), which is caused by germline loss-of-function CaSR mutations, did not reveal any alterations in glucose tolerance or insulin secretion (13). However, it may be that gain-of-function CaSR mutations, which cause autosomal dominant hypocalcemia (ADH) (14), are associated with abnormalities of glucose homeostasis and not FHH-associated loss-of-function CaSR mutations. To investigate this possibility, we have evaluated glucose tolerance and pancreatic islet function in a mouse model for ADH due to a germline gain-of-function CaSR mutation, Leu723Gln, referred to as *Nuclear flecks* (*Nuf*) because the mouse was initially identified to have cataracts (15, 16). Our analysis of these *Nuf* mice has demonstrated a role for the CaSR in glucose homeostasis.

## Materials and Methods

### Animals

All study animals were littermates aged between 20 and 28 weeks and kept in accordance with Home Office welfare guidance in an environment controlled for light (12 hours light and dark cycle), temperature (21°C ± 2°C), and humidity (55% ± 10%) at the Medical Research Council Harwell Centre. Mice had free access to water (25 ppm chlorine) and were fed *ad libitum* on a commercial diet (RM3; Special Diet Services, Essex, United Kingdom) that contained 1.24% calcium, 0.83% phosphorus, and 2948 IU/kg vitamin D. *Nuf* mice were maintained on the inbred 102/H background, which is a substrain bred at the Mary Lyon Centre (Harwell, United Kingdom) (15, 16). Animal studies were carried out in accordance with GlaxoSmithKline policy on the care, welfare, and treatment of animals, approved by the Medical Research Council Harwell Institute Ethical Review Committee, and licensed under the Animal (Scientific Procedures) Act 1986, issued by the UK Government Home Office Department (PPL30/2752).

### Compounds

Ronacaleret, which is also known as SB-751689, was provided by GlaxoSmithKline (London, United Kingdom) and dissolved in a 20% aqueous solution of 2-hydroxypropyl-*β*-cyclodextrin (catalog no. H107; Sigma-Aldrich, St. Louis, MO) prior to use in *in vitro* and *in vivo* studies.

### Cell culture and transfection

Human embryonic kidney (HEK) 293 cells were cultured in high-glucose Dulbecco’s modified Eagle medium (Invitrogen, Carlsbad, CA) supplemented with 10% fetal bovine serum, as described (17). WT (Leu723) and mutant (Gln723) CaSR-pEGFP-N1 constructs were generated, as reported (16), and transiently transfected into HEK293 cells using Lipofectamine Plus (Invitrogen), as described (16). Successful transfection of WT and mutant CaSR proteins was confirmed by visualizing green fluorescent protein (GFP) fluorescence using an Eclipse E400 fluorescence microscope with an epifluorescence filter, and images were captured using a DXM1200C digital camera and NIS Elements software (Nikon, Tokyo, Japan), as described (17).

### Measurement of Ca^2+^_i_ responses

The effect of ronacaleret on the Ca^2+^_i_ responses of CaSR-expressing cells was assessed by a flow cytometry-based assay, as reported (17, 18). In brief, 48 hours after transfection, the cells were harvested, washed in calcium- and magnesium-free Hank’s balanced salt solution (Invitrogen), and loaded with 1 μg/mL indo-1-acetoxymethylester (Molecular Probes, Eugene, OR) for 1 hour at 37°C (17, 18). After the removal of free dye, the cells were resuspended in calcium- and magnesium-free Hank’s balanced salt solution and maintained at 37°C. Transfected cells were incubated with either a 20% aqueous solution of 2-hydoxypropyl-*β*-cyclodextrin (vehicle) or ronacaleret at concentrations of 20 and 40 nM for 1 hour, as described (18). Flow cytometry was performed using a Beckman Coulter MoFlo XDP equipped with a JDSUY Xcyte ultraviolet laser and a Coherent Sapphire 488 laser with a 550LP dichroic mirror and 580/30 bandpass filter (17). Single cells were isolated and stimulated by sequentially adding calcium to increase the Ca^2+^_o_ concentration ([Ca^2+^]_o_) in a stepwise manner from 0 to 15 mM. The baseline fluorescence ratio was measured for 2 minutes, the fluorescence ratio compared with the time was recorded, and data were collected for 2 minutes at each [Ca^2+^]_o_, as described (17, 18). Cytomation Summit software was used to determine the peak mean fluorescence ratio of the transient response after each individual stimulus, which was expressed as a percentage of normalized response (17, 18). Nonlinear regression of the concentration-response curves was performed with GraphPad Prism to calculate the half-maximal effective concentration (EC_50_) responses for each separate experiment (17).

### Effect of ronacaleret on the glucose tolerance of Nuf mice

Ronacaleret (20 mg/mL) or drug vehicle was administered by twice daily oral gavage to mice over a 5-day period. The mice were then tested using the international mouse phenotyping consortium glucose tolerance test protocol (www.mousephenotype.org/impress/protocol/87/7). Briefly, mice were fasted for 16 hours, and a blood sample was obtained before intraperitoneal (IP) administration of a 2-g/kg glucose load. Subsequent blood samples were taken at 30, 60, and 120 minutes for plasma glucose and glucagon measurements or at 10, 20, and 30 minutes for plasma insulin measurements, as described (19).

### Body composition analysis

Fat and lean body mass of nonanesthetized live mice were measured using the Echo-MRI Analyzer system (Echo Medical Systems, Houston, TX), as described (20).

### Islet insulin and glucagon secretion

Pancreatic islets were isolated from whole mouse pancreata by collagenase digestion and separated from the suspension, as described (19). Islets were used for secretion experiments within 2 hours of isolation. Batches of 13 size-matched islets were incubated for 1 hour at 37°C in 0.3 mL of modified Krebs-Ringer buffer containing 2 mg/mL bovine serum albumin, 1.6 mM CaCl_2_, and 3 mM glucose, followed by a 1-hour incubation in 0.3 mL of the same Krebs-Ringer buffer supplemented with 1, 6, or 20 mM glucose, as described (21). A Krebs-Ringer buffer containing 0.8 mM CaCl_2_ was used to evaluate the effect of lowering the [Ca^2+^]_o_ on islet hormone secretion. The supernatant was used for measurement of secreted insulin and glucagon, and islets were lysed in cold acid ethanol for measurement of insulin and glucagon content. Insulin and glucagon were determined by radioimmunoassay (Millipore UK Ltd, Livingstone, United Kingdom) or using a rat/mouse insulin and glucagon duplex enzyme-linked immunosorbent assay (ELISA; Meso Scale Discovery, Rockville, MD).

### Quantitative reverse transcription polymerase chain reaction

Total RNA from isolated islets was extracted using an RNeasy Mini Kit (Qiagen, Hilden, Germany), and complementary DNA was generated by the Superscript II enzyme (Invitrogen), as described (19). QuantiTect primer assays were used to amplify selected genes (*Arx*, *Ccnd2*, *Foxm1*, *Foxo1*, *Irx2*, *Nkx6*, *Pax4*, *Pax6*, *Pdx1*, and *Tcf7l2*), which were analyzed by quantitative reverse transcription polymerase chain reaction (qRT-PCR) using SYBR Green (Qiagen) on the StepOnePlus qRT-PCR system (Life Technologies, Carlsbad, CA), as described (22). The *ΔΔ*Ct method was used to calculate fold change alterations in gene expression, relative to a housekeeping panel comprising the *Actb*, *Eef1b2*, and *Gapdh* genes (22).

### Biochemical analysis

Blood samples were collected from the lateral tail vein of study mice following application of topical local anesthesia, as reported (23), or collected from the retro-orbital vein under isoflurane terminal anesthesia. Plasma was separated by centrifugation at 5000*g* for 10 minutes at 8°C and analyzed for calcium and albumin on a Beckman Coulter AU680 analyzer, as described (15). Plasma calcium was adjusted for variations in albumin concentrations using the formula: plasma calcium (mmol/L) – **(**[plasma albumin (g/L) – 30] × 0.02**)**, as reported (23). Plasma glucose concentrations were measured using an Analox GM9 analyzer, as described (19). Plasma insulin concentrations were measured using a rat/mouse insulin ELISA (Millipore, Billerica, MA), as described (19), and plasma glucagon concentrations were measured using a rat/mouse glucagon ELISA (Mercodia, Uppsala, Sweden).

### Islet electrophysiology

Electrical activity was measured from *α*- and *β*-cells within intact mouse islets using the perforated-patch technique, as described (24), and all measurements were obtained at 34°C. Islets were immobilized using a wide-bore glass suction pipette (24) and perfused with modified Krebs-Ringer solution (140 mM NaCl, 3.6 mM KCl, 1.5 mM CaCl_2_, 0.5 mM MgSO_4_, 10 mM HEPES, 0.5 mM NaH_2_PO_4_, and NaHCO_3_ at pH 7.4 with NaOH and glucose as indicated), as reported (25). A Krebs-Ringer solution containing 0.75 mM CaCl_2_ was used to evaluate the effect of lowering the [Ca^2+^]_o_ on islet electrical activity. The solution within the pipet contained 76 mM K_2_SO_4_, 10 mM KCl, 10 mM NaCl, 1 mM MgCl_2_, and 5 mM HEPES (pH 7.35 using KOH) (24). To perforate the cell membrane, amphotericin B (6 μg/mL) was added to the intracellular buffer. The conductance of the *β*-cell adenosine triphosphate (ATP)–sensitive K^+^ (K_ATP_) channel within intact islets was measured using the perforated patch-clamping technique following exposure to different glucose concentrations or to tolbutamide (24). During the K_ATP_ channel conductance studies, *β*-cells were held at –70 mV, and K^+^ currents were evoked by exposing the cells to alternating 50 ms pulses of –60 or –80 mV (25). Islet cell types were established by their electrical activity in response to glucose, and cells that were electrically active at 1 mM glucose were identified as *α*-cells (26). Furthermore, *β*-cells were distinguished from non-*β*-cells by the absence of a voltage-gated Na^+^ current when a transient pulse from –70 to 0 mV was applied (21). Measurements were undertaken in individual islets using an EPC-10 patch-clamp amplifier (HEKA Electronics, Ludwigshafen/Rhein, Germany) and Pulse software (version 8.50), as described previously (24).

### Islet area analysis

Mouse pancreata were fixed in 10% neutral buffered formalin, mounted longitudinally, and paraffin embedded, as described (19). Serial sections (4.5 µm) were cut, and every 10th section stained with hematoxylin and eosin (H&E), as described (19). Images of ≥10 H&E-stained sections per mouse were acquired at a ×20 magnification using the semiautomated TissueFax slide-scanning microscope (TissueGnostics, Vienna, Austria), as described (27). Islets were identified, and islet area, size, and number were quantified using HistoQuest software (TissueGnostics) (27). Islet area was normalized to the total section area and to body weight, and islet size was calculated by dividing the total islet area per section by the number of islets on the same section.

### Islet immunohistochemistry

Immunohistochemistry was undertaken using paraffin-embedded pancreatic sections that had been subjected to heat-induced epitope retrieval in citrate buffer (pH 6.0), followed by blocking in 10% donkey serum for 1 hour. Primary antibodies used for insulin, glucagon, and Ki-67 staining were guinea pig anti-insulin **(**1:200; ab7842, Abcam [research resource identifier (RRID): AB_306130]**)**, rabbit anti-glucagon [1:200; ab92517, Abcam (RRID: AB_10561971)], and rabbit anti-Ki67 [1:500; ab15580, Abcam (RRID: AB_443209)], respectively. Secondary antibodies used were donkey anti–guinea pig [706-225-148, Cy2, Jackson (RRID: AB_2340467)] 1:100 and donkey anti-rabbit [711-165-152, Cy3, Jackson (RRID: AB_2307443)] 1:500 in phosphate-buffered saline. Sections were mounted in ProLong Gold Antifade Reagent containing 4′,6-diamidino-2-phenylindole (DAPI; Life Technologies). Images of whole sections were acquired using the TissueFax slide-scanning microscope (TissueGnostics), as described (27). Quantification of immunofluorescence signals was undertaken using the semiautomated intensity detection function of the TissueQuest software (TissueGnostics), as described (27). The numbers of *α*- and *β*-cells within individual islets were quantified using the cell-based analysis profile of the TissueQuest software (27), normalized to the total islet area, and reported as percentage of the mean numbers of *Casr^+/+^* *α*- and *β*-cells, respectively.

### Statistical analysis

The *in vitro* studies involved two separate transfection experiments and eight to nine technical assays. Statistical comparisons of the EC_50_ responses were undertaken using the *F* test (17, 18). For the *in vivo* and *ex vivo* studies, the Mann-Whitney *U* test was used to compare differences between two groups, and the Kruskal-Wallis test was used to compare multiple groups. An unpaired Student *t* test was used to compare groups with small sample sizes (n < 5), as reported (28). All analyses were undertaken using GraphPad Prism (GraphPad), and a value of *P* < 0.05 was considered significant for all analyses.

## Results

### *Nuf* mice have impaired glucose tolerance that is ameliorated by a CaSR allosteric modulator

**Figure 1.**
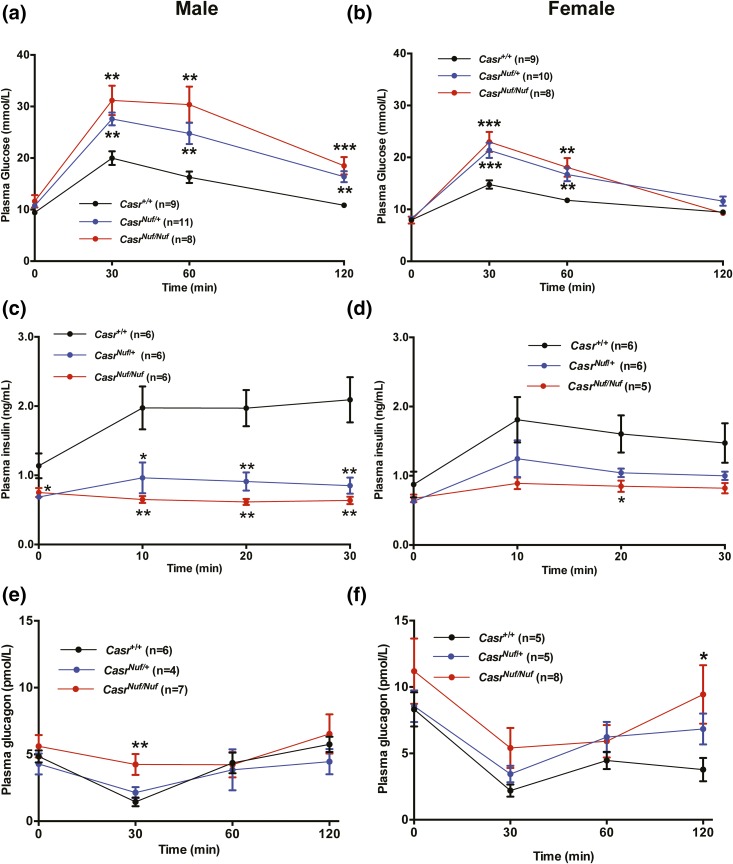
Plasma glucose, insulin, and glucagon concentrations during IPGTT testing. (a) Male and (b) female *Casr^Nuf/+^* (blue) and *Casr^Nuf/Nuf^* mice (red) are significantly hyperglycemic compared with respective *Casr*^+/+^ mice (black) during a 2-hour IPGTT. (c) Male and (d) female *Casr^Nuf/Nuf^* mice and male *Casr^Nuf/+^* mice have significantly reduced plasma insulin concentrations compared with respective *Casr^+/+^* mice during a 30-minute IPGTT. (e) Male and (f) female *Casr^Nuf/Nuf^* mice show significant elevations in plasma glucagon concentrations compared with respective *Casr^+/+^* mice during a 2-hour IPGTT. Results are expressed as mean ± standard error of the mean. *P < 0.05, **P < 0.01, and ***P < 0.001 compared with *Casr^+/+^* mice at respective time points.

To establish whether the gain-of-function CaSR mutation in *Nuf* mice may be associated with alterations in glucose homeostasis, IP glucose tolerance testing (IPGTT) was performed on WT (*Casr^+/+^*), heterozygous- (*Casr^Nuf/+^*), and homozygous-affected (*Casr^Nuf/Nuf^*) mice aged 20 to 28 weeks that had been fasted for 16 hours. Plasma glucose concentrations were measured at 0, 30, 60, and 120 minutes following an IP 2-g/kg glucose bolus injection. Male and female *Casr^Nuf/+^* and *CasrCasr^Nuf/Nuf^* mice had elevated plasma glucose concentrations at 30 and 60 minutes, which were significantly (*P* < 0.01) greater than those of respective *Casr^+/+^* mice [Fig. 1(a) and 1(b)]. The impaired glucose tolerance was not associated with any alterations in body weight or in fat or lean mass (Supplemental Fig. 1). To test whether the impaired glucose tolerance of *Nuf* mice may be associated with abnormalities of insulin secretion *in vivo*, an IPGTT was conducted with plasma samples collected for insulin measurement at 0, 10, 20, and 30 minutes. Plasma insulin concentrations of male and female *Casr^+/+^* mice increased twofold at 10 minutes after an IP 2 g/kg glucose bolus injection [Fig. 1(c) and 1(d)]. However, male affected *Casr^Nuf/+^* and *Casr^Nuf/Nuf^* mice showed significantly reduced plasma insulin concentrations at 10 and 20 minutes following glucose administration [Fig. 1(c)], whereas only female *Casr^Nuf/Nuf^* mice showed significantly reduced insulin concentrations at 20 minutes [Fig. 1(d)] compared with respective *Casr^+/+^* mice. Affected male *Casr^Nuf/Nuf^* mice were also shown to have an inadequate suppression of plasma glucagon concentrations at the 30-minute time point during a 120-minute IPGTT [Fig. 1(e)], whereas female *Casr^Nuf/Nuf^* mice had significantly raised plasma glucagon concentrations at 120 minutes [Fig. 1(f)]. No significant differences in the glucose, insulin, or glucagon responses were noted between male and female mice (Supplemental Fig. 2).

**Figure 2.**
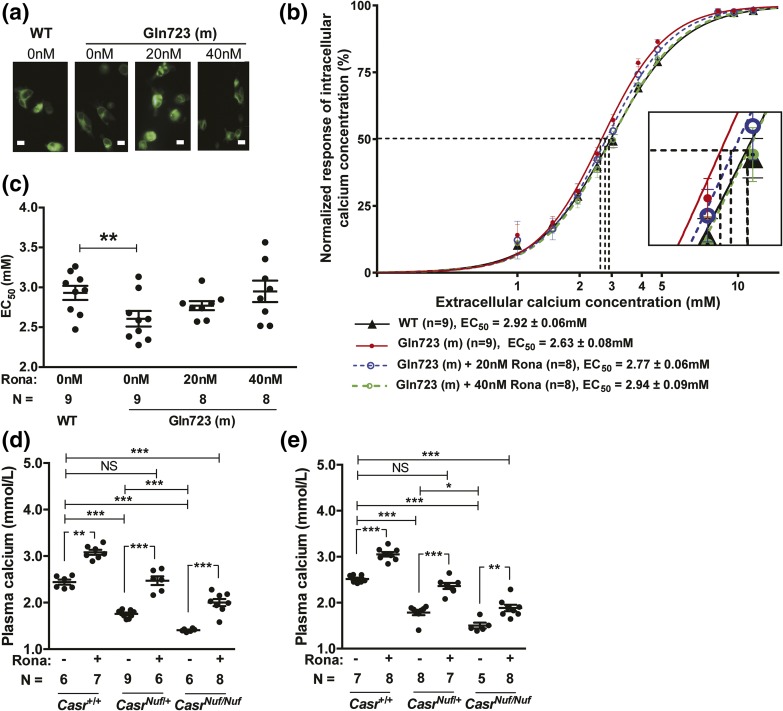
Effect of ronacaleret on the CaSR gain-of-function and hypocalcemia of *Nuf* mice. (a) Fluorescence microscopy of HEK293 cells transiently transfected with WT Leu723 or mutant (m) Gln723 CASR-pEGFP-N1 constructs. GFP expression in these cells indicates successful transfection and expression by these constructs. Bar indicates 10 μm. (b) Effect of ronacaleret treatment on the intracellular calcium responses of the Gln723 CaSR mutant. The Gln723 CaSR mutant led to a leftward shift in the concentration-response curve (solid red line) compared with the WT (Leu723) CaSR (solid black line). The addition of ronacaleret (Rona) at 20- and 40-nM concentrations rectified the leftward shift of the Gln723 CaSR mutant (blue dashed line and green dashed line, respectively). The zoomed-in image shows the concentration-response curves at the EC_50_ values of the WT and mutant CaSRs. (c) Effect of 20 and 40 nM ronacaleret on the EC_50_ values of the Gln723 CaSR mutant. (d) Male and (e) female *Casr^Nuf/+^* and *Casr^Nuf/Nuf^* mice were significantly hypocalcemic compared with respective *Casr^+/+^* mice. Treatment with 90-mg/kg ronacaleret significantly increased plasma calcium concentrations in Casr^+/+^, *Casr^Nuf/+^*, and *Casr^Nuf/Nuf^* mice compared with respective mice treated with the drug vehicle only. Ronacaleret treatment normalized the plasma calcium concentrations of *Casr^Nuf/+^* mice. However, the plasma calcium concentrations of treated *Casr^Nuf/Nuf^* mice remained significantly reduced compared with untreated *Casr^+/+^* mice. Mean ± standard error of the mean values are indicated by solid bars. *P < 0.05, **P < 0.01, ***P < 0.001. NS, nonsignificant.

**Figure 3.**
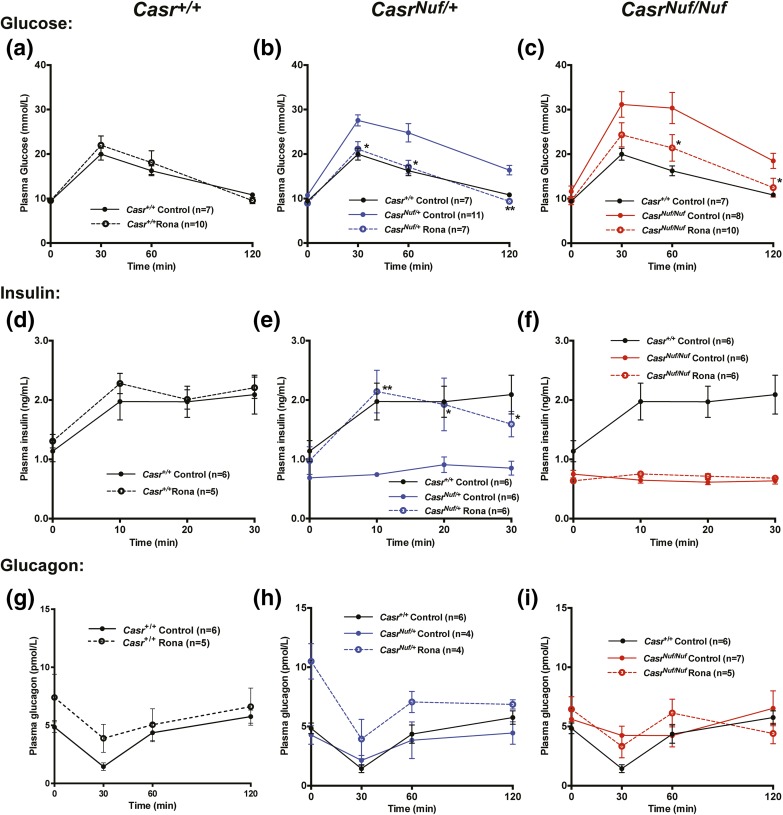
Effect of ronacaleret on the plasma glucose, insulin, and glucagon concentrations of male mice during IPGTT. Ronacaleret administration had no effect on the plasma glucose concentrations of (a) *Casr^+/+^* mice (black dashed line) but significantly lowered plasma glucose in (b) *Casr^Nuf/+^* (blue dashed line) and (c) *Casr^Nuf/Nuf^* mice (red dashed line) compared with respective control mice treated with the drug vehicle only (represented by solid lines), so that the glucose concentrations were not significantly different from *Casr^+/+^* mice. Ronacaleret had no effect on the plasma insulin concentrations of (d) *Casr^+/+^* mice but significantly increased plasma insulin in (e) *Casr^Nuf/+^* mice compared with controls, so that the insulin concentrations were not significantly different from *Casr^+/+^* mice. Ronacaleret treatment did not alter plasma insulin concentrations in (f) *Casr^Nuf/Nuf^* mice. Ronacaleret had no significant effect on the plasma glucagon concentrations of (g) *Casr^+/+^* mice, (h) *Casr^Nuf/+^* mice, or (i) *Casr^Nuf/Nuf^* mice compared with respective control mice. Results are expressed as mean ± standard error of the mean. *P < 0.05, **P < 0.01 compared with control mice.

**Figure 4.**
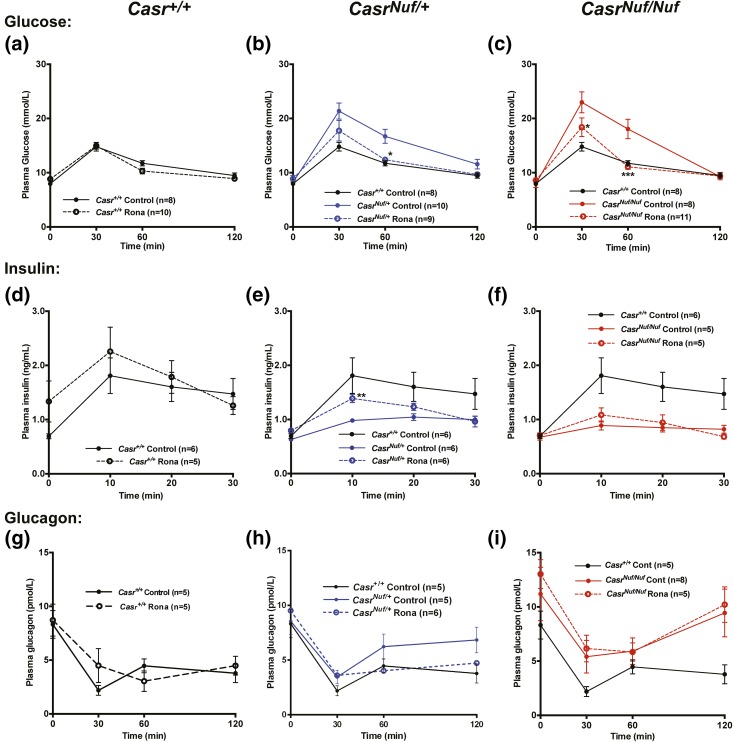
Effect of ronacaleret on the plasma glucose, insulin, and glucagon concentrations of female mice during IPGTT. Ronacaleret administration had no effect on the plasma glucose concentrations of (a) *Casr^+/+^* mice (black dashed line) but significantly lowered plasma glucose in (b) *Casr^Nuf/+^* (blue dashed line) and (c) *Casr^Nuf/Nuf^* mice (red dashed line) compared with respective control mice treated with the drug vehicle only (represented by solid lines), so that the glucose concentrations were not significantly different from *Casr^+/+^* mice. Ronacaleret had no effect on the plasma insulin concentrations of (d) *Casr^+/+^* mice but significantly increased plasma insulin in (e) *Casr^Nuf/+^* mice compared with controls, so that the insulin concentrations were not significantly different from *Casr^+/+^* mice. Ronacaleret treatment did not alter plasma insulin concentrations in (f) *Casr^Nuf/Nuf^* mice. Ronacaleret had no significant effect on the plasma glucagon concentrations of (g) *Casr^+/+^* mice, (h) *Casr^Nuf/+^* mice, or (i) *Casr^Nuf/Nuf^* mice compared with respective control mice. Results are expressed as mean ± standard error of the mean. *P < 0.05, **P < 0.01, ***P < 0.001 compared with control mice.

To investigate if the impaired glucose tolerance of *Nuf* mice, which have the Leu723Gln gain-of-function CaSR mutation, could be corrected by a selective CaSR negative allosteric modulator (*i.e.*, calcilytic agent), we assessed the *in vitro* and *in vivo* effects of ronacaleret, a calcilytic compound (29). For the *in vitro* studies, HEK293 cells were transiently transfected with WT (Leu723) or mutant (Gln723) *CASR*-pEGFP-N1 constructs, which express the CaSR protein fused to the N terminus of enhanced GFP (16), and the effect of ronacaleret on the responses of Ca^2+^_i_ concentrations to alterations in [Ca^2+^]_o_ was assessed. HEK293 cells expressing the mutant Gln723 CaSR [Fig. 2(a)] were shown to have a leftward shift of the concentration-response curve [Fig. 2(b)], with a significant reduction in EC_50_ (2.63 ± 0.08 mM) compared with WT (2.92 ± 0.06 mM; *P* < 0.01) [Fig. 2(c)], consistent with a gain-of-function, as reported (16). A dose titration of ronacaleret revealed 20 and 40 nM concentrations of this calcilytic compound to normalize the EC_50_ values and shift in the concentration-response curve of mutant Gln723-expressing cells [Fig. 2(b) and 2(c)]. Glucose has recently been reported to lead to allosteric activation of the CaSR (30), and we investigated the effect of alterations in glucose concentrations on the Ca^2+^_i_ responses of WT and *Nuf* mutant Gln723 CaSRs, which were stably expressed in HEK293 cells (Supplemental Fig. 3). Our findings showed that altering the glucose concentration from 3 to 25 mM had no effect on the EC_50_ values of cells stably expressing WT or *Nuf* mutant Gln723 CaSRs, whereas the addition of 40 nM ronacaleret significantly increased the EC_50_ values of these cells (Supplemental Fig. 3). To determine whether amelioration of CaSR gain-of-function by ronacaleret may lead to an improvement in glucose tolerance *in vivo*, we administered this calcilytic agent to *Nuf* mice. Male and female *Casr^+/+^*, *Casr^Nuf/+^*, and *Casr^Nuf/Nuf^* mice were given ronacaleret or drug vehicle for 5 days by twice-daily oral gavage. Ronacaleret was administered at a dose of 90 mg/kg, as pilot studies had shown this dose to increase plasma calcium concentrations and to be well tolerated in *Casr^+/+^* mice. Untreated *Casr^Nuf/+^* and *Casr^Nuf/Nuf^* mice were shown to be significantly hypocalcemic compared with *Casr^+/+^* mice, and *Casr^Nuf/Nuf^* mice had significantly lower plasma calcium concentrations than *Casr^Nuf/+^* mice [Fig. 2(d) and 2(e)]. Ronacaleret treatment significantly (*P* < 0.01) increased plasma calcium concentrations in male and female *Casr^+/+^*, *Casr^Nuf/+^*, and *Casr^Nuf/Nuf^* mice compared with respective untreated mice [Fig. 2(d) and 2(e)]. Ronacaleret treatment normalized the plasma calcium concentrations of male and female *Casr^Nuf/+^* mice [Fig. 2(d) and 2(e)]. However, the plasma calcium concentrations of treated *Casr^Nuf/Nuf^* mice remained significantly reduced compared with untreated *Casr^+/+^* mice [Fig. 2(d) and 2(e)]. Ronacaleret treatment had no effect on the plasma glucose concentrations of male and female *Casr^+/+^* mice (Figs. 3 and 4) but significantly (*P* < 0.05) improved glucose tolerance in male and female *Casr^Nuf/+^* and *Casr^Nuf/Nuf^* mice compared with respective mice treated with the drug vehicle alone (Figs. 3 and 4). Moreover, sex differences were noted, as ronacaleret normalized plasma glucose concentrations at 30 minutes in male *Casr^Nuf/+^*mice but only at 60 minutes in female *Casr^Nuf/+^*mice (Figs. 3 and 4). Ronacaleret treatment had no effect on the plasma insulin concentrations of male and female *Casr^+/+^* mice (Figs. 3 and 4) but significantly increased the plasma insulin concentrations of male and female *Casr^Nuf/+^* mice compared with untreated *Casr^Nuf/+^* mice (Figs. 3 and 4). Ronacaleret treatment did not alter plasma insulin concentrations in male and female *Casr^Nuf/Nuf^* mice (Figs. 3 and 4) and had no significant effect on the plasma glucagon concentrations of male and female *Casr^+/+^*, *Casr^Nuf/+^*, or *Casr^Nuf/Nuf^* mice (Figs. 3 and 4). No significant differences were noted between the biochemical responses of ronacaleret-treated male and female mice (Supplemental Fig. 3). To evaluate the mechanisms underlying these alterations of glucose tolerance and plasma insulin and glucagon concentrations in *Nuf* mice, further *ex vivo* and electrophysiological studies were undertaken. As no significant differences had been observed for the glucose, insulin, and glucagon responses of male and female mice, the *ex vivo* data were combined for males and females.

### Pancreatic islet size and proliferation

**Figure 5.**
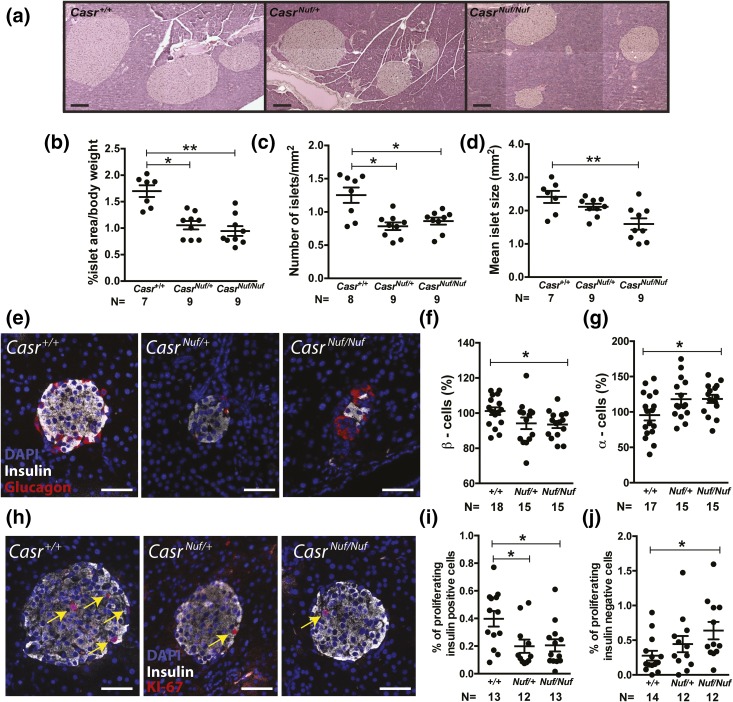
Histological analysis of *Nuf* mice pancreatic islets. (a) Representative H&E-stained pancreatic sections from *Casr^+/+^*, *Casr^Nuf/+^*, and *Casr^Nuf/Nuf^* mice. Bars indicate 200 μm. (b) Islet area and (c) number are significantly reduced in *Casr^Nuf/+^* and *Casr^Nuf/Nuf^* mice compared with *Casr^+/+^* mice. (d) Islet size is significantly reduced in *Casr^Nuf/Nuf^* mice compared with *Casr^+/+^* mice. (e) Representative pancreatic islets from *Casr^+/+^*, *Casr^Nuf/+^*, and *Casr^Nuf/Nuf^* mice immunostained for glucagon (red), insulin (white), and DAPI (blue). Bars indicate 50 μm. (f) *Casr^Nuf/Nuf^* mice have significantly reduced *β*-cell numbers and (g) significantly increased *α*-cell numbers compared with *Casr^+/+^* mice. (h) *β*-cell proliferation in representative islets from *Casr^+/+^*, *Casr^Nuf/+^*, and *Casr^Nuf/Nuf^* mice immunostained for insulin (white), DAPI (blue), and KI-67 (red). KI-67–positive cells are also indicated by yellow arrows. Bars indicate 50 μm. (i) *Casr^Nuf/Nuf^* mice have significantly reduced proliferation of *β*-cells and (j) significantly increased proliferation of *α*-cells compared with respective *Casr^+/+^* mice. Results are expressed as mean ± standard error of the mean. *P < 0.05, **P < 0.01 compared with *Casr^+/+^* mice.

We assessed for alterations in islet morphology by undertaking histological analysis of whole pancreases from adult *Casr^+/+^*, *Casr^Nuf/+^*, and *Casr^Nuf/Nuf^* mice. This revealed that the overall architecture of *Casr^Nuf/+^* and *Casr^Nuf/Nuf^* islets was similar to that in *Casr^+/+^* mice [Fig. 5(a)]. However, islet area, which was normalized to body weight, was reduced by >40% in *Casr^Nuf/+^* and *Casr^Nuf/Nuf^* mice [Fig. 5(b)], and this was associated with significant decreases in islet numbers and mean islet size [Fig. 5(c) and 5(d)]. To assess whether the reduced islet area may also be associated with alterations in the numbers of *β*-cells or *α*-cells, whole pancreas sections were immunostained for insulin and glucagon [Fig. 5(e)]. Individual islets from *Casr^Nuf/Nuf^* mice had 5% to 10% fewer *β*-cells (*P* < 0.05) and approximately 20% more *α*-cells than *Casr^+/+^* islets (*P* < 0.05) [Fig. 5(f) and 5(g)]. To investigate whether the reduction in *β*-cells and increase in *α*-cells were caused by alterations in cellular proliferation, whole pancreas sections were immunostained with the proliferation marker Ki-67. [Fig. 5(h)]. The percentage of proliferating insulin-positive *β*-cells in *Casr^Nuf/Nuf^* mice was found to be significantly decreased (*P* < 0.05), whereas the percentage of proliferating insulin-negative cells (which are predominantly *α*-cells) was significantly increased when compared with respective *Casr^+/+^* islets [Fig. 5(i) and 5(j)]. qRT-PCR analysis utilizing RNA from isolated *Casr^+/+^* and *Casr^Nuf/Nuf^* islets revealed that these changes in *β*-cell and *α*-cell proliferation were not associated with significant alterations in the expression of genes regulating islet mass such as *Foxo1*, *Foxm1*, *Ngn3*, and *Tcf7l2* (31–34), which promote *β*-cell proliferation, or in the expression of genes such as *Arx* and *Irx2* (35), which influence *α*-cell proliferation (Supplemental Fig. 4).

### Insulin and glucagon secretion from isolated islets

**Figure 6.**
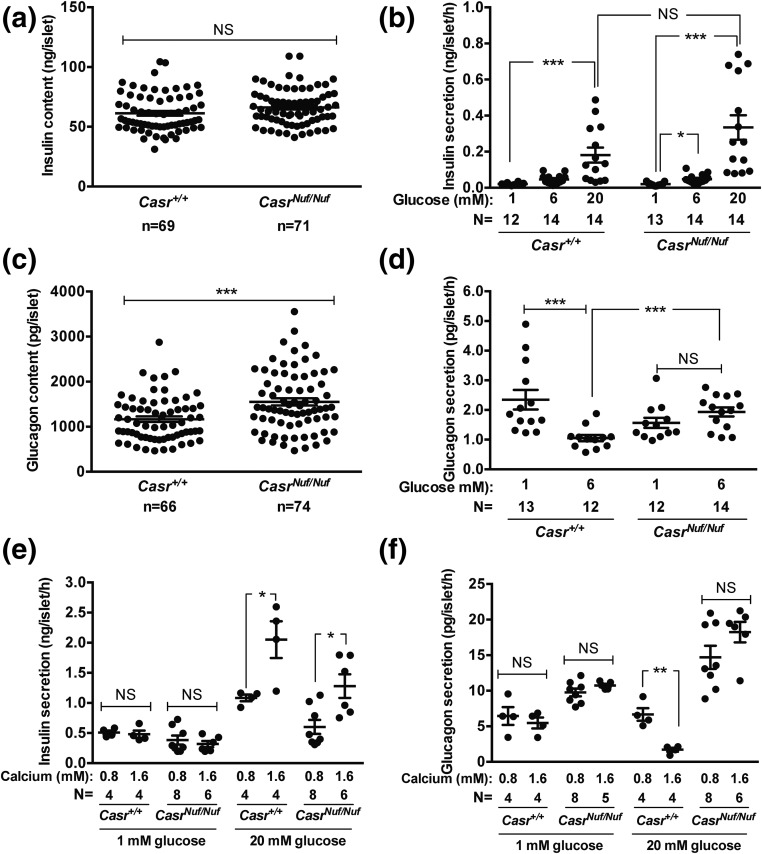
Insulin and glucagon secretion from isolated *Nuf* mice pancreatic islets. (a) The total insulin content of *Casr^Nuf/Nuf^* islets was not altered compared with *Casr^+/+^* islets. (b) *Casr^+/+^* and *Casr^Nuf/Nuf^* islets were incubated in 1.6 mM [Ca^2+^]_o_ and exposed to varying glucose concentrations (1, 6, or 20 mM). *Casr^+/+^* and *Casr^Nuf/Nuf^* islets showed significantly increased insulin secretion following stimulation with 20 mM glucose. No significant differences in the maximal insulin secretory responses were observed between *Casr^+/+^* and *Casr^Nuf/Nuf^* islets. (c) The total glucagon content of *Casr^Nuf/Nuf^* islets was significantly increased compared with *Casr^+/+^* islets. (d) *Casr^+/+^* and *Casr^Nuf/Nuf^* islets were incubated in 1.6 mM [Ca^2+^]_o_ and exposed to 1 and 6 mM glucose concentrations. *Casr^+/+^* islets showed a significant reduction in glucagon secretion following stimulation with 6 mM glucose. In contrast, glucagon secretion from *Casr^Nuf/Nuf^* islets failed to suppress following glucose stimulation, and *Casr^Nuf/Nuf^* islets had significantly increased glucagon secretion compared with *Casr^+/+^* islets at 6 mM glucose. (e) The effect of Ca^2+^_o_ on insulin secretion was assessed by incubating *Casr^+/+^* and *Casr^Nuf/Nuf^* islets with varying Ca^2+^_o_ concentrations (0.8 or 1.6 mM) and exposing them to low (1 mM) or high (20 mM) glucose. Exposure to low (0.8 mM) Ca^2+^_o_ suppressed insulin secretion from *Casr^+/+^* and *Casr^Nuf/Nuf^* islets at 20 mM glucose. (f) Exposure to low (0.8 mM) Ca^2+^_o_ increased glucagon secretion from *Casr^+/+^* islets at 20 mM glucose but had no effect on glucagon secretion from *Casr^Nuf/Nuf^* islets. Islet insulin and glucagon in panels (a–d) were measured by radioimmunoassay and by duplex rat/mouse ELISA (Meso Scale Discovery) in panels (e) and (f). The sample size (N) represents batches of size-matched islets, which were pooled from three to six *Casr^+/+^* mice and six *Casr^Nuf/Nuf^* mice. Mean ± standard error of the mean values for the respective groups are indicated by solid bars. *P < 0.05, **P < 0.01, ***P < 0.001. NS, nonsignificant.

To determine whether *Nuf* mice have alterations in pancreatic islet insulin secretion, size-matched islets were isolated from *Casr^+/+^* and *Casr^Nuf/Nuf^* mice and exposed to low (1 mM), physiological (6 mM), or high (20 mM) glucose concentrations in the presence of 1.6 mM [Ca^2+^]_o_, which represents a physiological [Ca^2+^]_o_ (36). The insulin content of isolated *Casr^Nuf/Nuf^* islets was not significantly different from isolated *Casr^+/+^* islets [Fig. 6(a)]. Measurement of insulin in the supernatant of islets following glucose stimulation did not reveal any impairment in the insulin secretory responses of isolated *Casr^Nuf/Nuf^* islets compared with *Casr^+/+^* islets [Fig. 6(b)]. We also investigated whether glucagon secretion may be altered in *Nuf* mouse islets. Compared with *Casr^+/+^* islets, there was a >30% increase in the glucagon content [Fig. 6(c)] of *Casr^Nuf/Nuf^* islets. Increasing glucose from 1 to 6 mM resulted in a 60% reduction of glucagon secretion from isolated *Casr^+/+^* islets [Fig. 6(d)]. In contrast, islets from *Casr^Nuf/Nuf^* mice exhibited a lack of glucose-induced suppression of glucagon release [Fig. 6(d)], which is consistent with that observed in islets from type 2 diabetic patients (37). To investigate whether the reduced plasma insulin concentrations of *Nuf* mice may have been a consequence of their hypocalcemia, insulin secretion from isolated islets was measured following exposure to 0.8 mM [Ca^2+^]_o_, which is similar to the plasma calcium concentrations observed in *Casr^Nuf/Nuf^* mice (15, 16). Altering the [Ca^2+^]_o_ had no effect on insulin secretion in the presence of low (1 mM) glucose concentrations [Fig. 6(e)]. However, exposure to low (0.8 mM) Ca^2+^_o_ impaired insulin secretion from *Casr^+/+^* and *Casr^Nuf/Nuf^* islets in the presence of high (20 mM) glucose concentrations [Fig. 6(e)]. Exposure to low (0.8 mM) Ca^2+^_o_ increased glucagon secretion from *Casr^+/+^* islets at 20 mM glucose but had no effect on glucagon secretion from *Casr^Nuf/Nuf^* islets [Fig. 6(f)].

#### Electrophysiological studies of isolated islets

**Figure 7.**
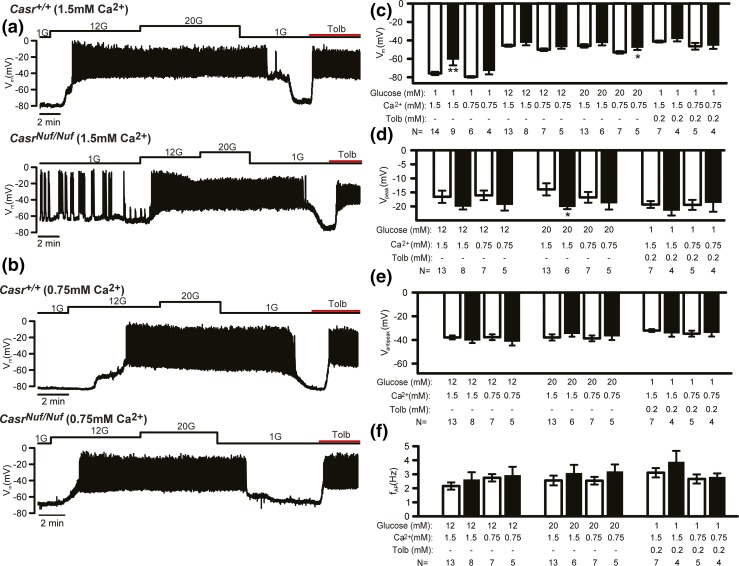
Effect of glucose stimulation on the electrical activity of *Nuf* mice *β*-cells. (a) Representative membrane potential recording of *β*-cells from intact *Casr^+/+^* and *Casr^Nuf/Nuf^* islets, which had been incubated in 1.5 mM [Ca^2+^]_o_ and following stimulation with 1 mM (1G), 12 mM (12G), and 20 mM (20G) glucose concentrations or with tolbutamide (Tolb). (b) Representative membrane potential recording of *β*-cells from intact *Casr^+/+^* and *Casr^Nuf/Nuf^* islets in the presence of 0.75 mM Ca^2+^_o_ concentrations and following stimulation with 1, 12, or 20 mM glucose concentrations or with tolbutamide. (c) Basal membrane potential, (d) action potential peak, (e) antipeak potential, and (f) frequency of action potential firing from *β*-cells was assessed in intact *Casr^+/+^* (white bars) and *Casr^Nuf/Nuf^* islets (black bars) in the presence of 1.5 or 0.75 mM Ca^2+^_o_ concentrations and following stimulation with glucose or tolbutamide. The sample size (N) represents individual *β*-cell recordings obtained from intact islets of six *Casr^+/+^* mice and four *Casr^Nuf/Nuf^* mice. Results are expressed as mean ± standard error of the mean. *P < 0.05, **P < 0.01 compared with *Casr^+/+^* mice at respective glucose and Ca^2+^_o_ concentrations.

**Figure 8.**
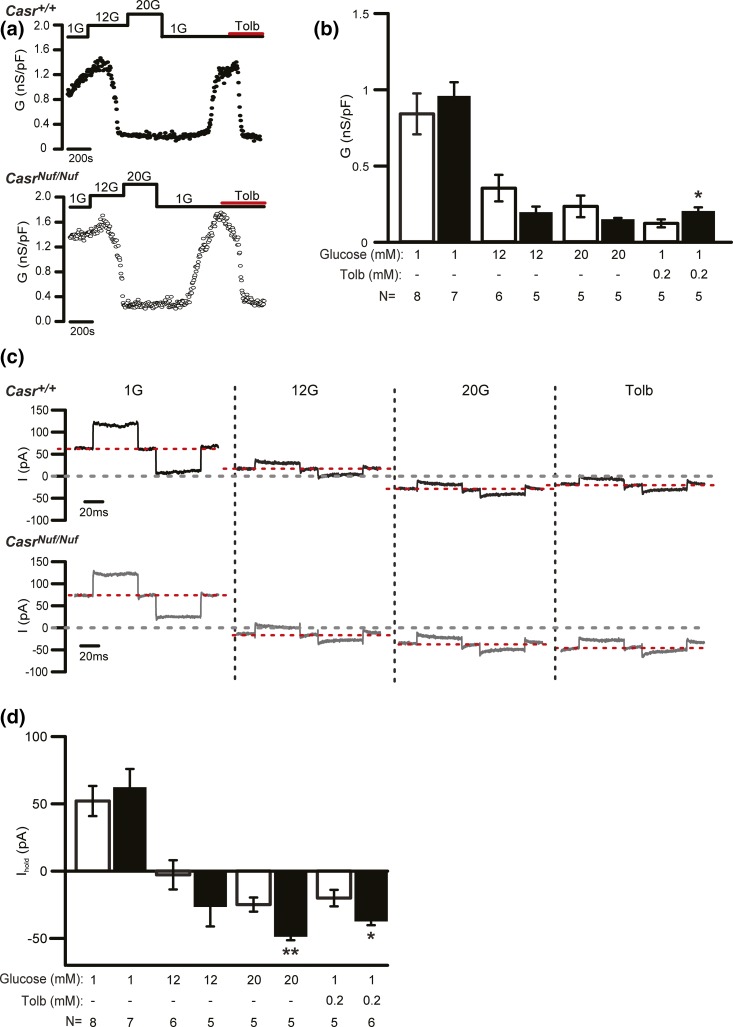
K_ATP_ channel conductance of *Nuf* mice *β*-cells. (a) Representative recording of *β*-cell K_ATP_ channel conductance from intact *Casr^+/+^* and *Casr^Nuf/Nuf^* islets after islets had been incubated in 1.5 mM [Ca^2+^]_o_ and following stimulation with 1 mM (1G), 12 mM (12G), and 20 mM (20G) glucose concentrations or with tolbutamide (Tolb). (b) Analysis of K_ATP_ channel conductance from *β*-cells within intact *Casr^+/+^* (white bars) and *Casr^Nuf/Nuf^* islets (black bars) following stimulation with glucose or tolbutamide. (c) Representative traces of *β*-cell background current measurement following glucose stimulation or treatment with tolbutamide. (d) Analysis of holding current from *β*-cells within intact *Casr^+/+^* (white bars) and *Casr^Nuf/Nuf^* islets (black bars) following stimulation with glucose or tolbutamide. The sample size (N) represents individual *β*-cell recordings obtained from intact islets of five *Casr^+/+^* mice and five *Casr^Nuf/Nuf^* mice. Results are expressed as mean ± standard error of the mean. *P < 0.05, **P < 0.01 compared with *Casr^+/+^* mice at respective glucose and tolbutamide concentrations.

We investigated alterations in *β*-cell electrical activity by recording the membrane potential of *β*-cells within intact *Casr^+/+^* and *Casr^Nuf/Nuf^* islets on treatment with varying (1, 12, or 20 mM) concentrations of glucose, or tolbutamide, which is a K_ATP_ channel blocker (26). The electrophysiological experiments were undertaken at 1.5 mM [Ca^2+^]_o_, as described (25), and the effect of lowering the [Ca^2+^]_o_ on *β*-cell electrical activity was evaluated at 0.75 mM [Ca^2+^]_o_, which is in keeping with the plasma calcium concentrations of *Casr^Nuf/Nuf^* mice (15, 16). Analysis of membrane potentials showed *β*-cells from *Casr^+/+^* mice (*i.e.*, WTs) to be hyperpolarized (–76 ± 2 mV) and electrically silent at 1 mM glucose concentrations [Fig. 7(a)]. In contrast, *Casr^Nuf/Nuf^* *β*-cells were significantly depolarized (–63 ± 5 mV; *P* < 0.01) at 1 mM glucose, and >40% of cells (four of nine) were electrically active with low-frequency action potential firing [Fig. 7(a)]. The depolarization and hyperactivity of *Casr^Nuf/Nuf^* *β*-cells were rectified by lowering [Ca^2+^]_o_ from 1.5 to 0.75 mM [Fig. 7(b) and 7(c)]. At stimulatory glucose concentrations (12 or 20 mM) or following application of tolbutamide, both *Casr^+/+^* and *Casr^Nuf/Nuf^* *β*-cells were depolarized and firing action potentials [Fig. 7(a) and 7(b)], and the level of the depolarization was not altered in *Casr^Nuf/Nuf^* *β*-cells [Fig. 7(c)]. However, the peak of action potential evoked by 20 mM glucose in *Casr^Nuf/Nuf^* *β*-cells was significantly reduced compared with *Casr^+/+^* *β*-cells [Fig. 7(d)]. The antipeak potential and firing frequency were not affected by the expression of the *Casr* mutation or variation in [Ca^2+^]_o_ [Fig. 7(e) and 7(f)]. Based on the observation that *Casr^Nuf/Nuf^* *β*-cells were significantly depolarized at 1 mM glucose, we postulated that the CaSR may influence the K_ATP_ channel, which plays a central role in regulating the membrane potential of *β*-cells (1). We therefore measured resting conductance, which predominantly reflects K_ATP_ channel activity, of *Casr^+/+^* and *Casr^Nuf/Nuf^* *β*-cells in the presence of 1, 12, or 20 mM glucose, or with tolbutamide. These studies showed *β*-cell resting conductance to be comparable between genotypes [Fig. 8(a) and 8(b)]. However, the holding current measured at –70 mV in *Casr^Nuf/Nuf^* *β*-cells when K_ATP_ channel activity was suppressed by 20 mM glucose or tolbutamide was significantly greater than in *Casr^+/+^* *β*-cells [Fig. 8(c) and 8(d)], and its contribution likely accounts for the more depolarized membrane potential and action potential firing in *Casr^Nuf/Nuf^* *β*-cells exposed to 1 mM glucose [Fig. 7(a)].

**Figure 9.**
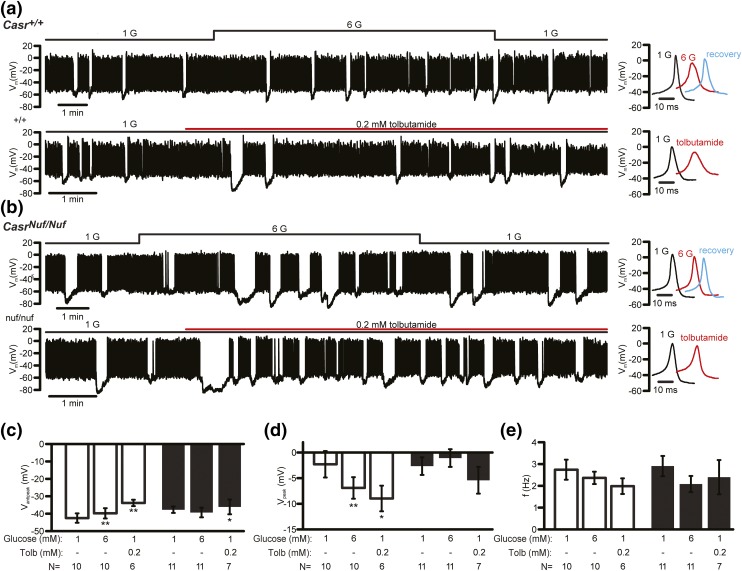
Effect of glucose stimulation on the electrical activity of *Nuf* mice *α*-cells. Representative membrane potential recording of *α*-cells from (a) intact *Casr^+/+^* and (b) *Casr^Nuf/Nuf^* islets, which had been incubated in 1.5 mM [Ca^2+^]_o_ and following stimulation with 1 mM (1G) and 6 mM (6G) glucose concentrations or with tolbutamide (Tolb). (c) Antipeak potential, (d) action potential peak, and (e) frequency of action potential firing was assessed in *α*-cells within intact *Casr^+/+^* (white bars) and *Casr^Nuf/Nuf^* islets (black bars) following stimulation with glucose or tolbutamide. The sample size (N) represents individual *α*-cell recordings obtained from intact islets of five *Casr^+/+^* mice and seven *Casr^Nuf/Nuf^* mice. Results are expressed as mean ± standard error of the mean. *P < 0.05, **P < 0.01 compared with respective *α*-cells at 1 mM glucose.

To determine whether *Nuf* mice may also have alterations in *α*-cell electrical activity, membrane potentials were recorded in intact islet *α*-cells, as described (26). In agreement with previous reports (37), *Casr^+/+^* *α*-cells were shown to be electrically active at 1 mM glucose [Fig. 9(a)]. The addition of 6 mM glucose led to a small but statistically significant (*P* < 0.01) depolarization and reduction in action potential peak [Fig. 9(a)]. *Casr^Nuf/Nuf^* *α*-cells were also electrically active at 1 mM glucose [Fig. 9(b)] but did not depolarize when glucose was increased to 6 mM [Fig. 9(b) and 9(c)], and there was no change in the action potential peak [Fig. 9(b) and 9(d)]. The addition of tolbutamide led to membrane depolarization in both *Casr^+/+^* and *Casr^Nuf/Nuf^* *α*-cells [Fig. 9(a) and 9(b)], but the magnitude of the depolarizing effect was reduced in *Casr^Nuf/Nuf^* *α*-cells [Fig. 9(c)]. Tolbutamide also decreased the action potential peak of *Casr^+/+^* *α*-cells but had no significant effect on the action potential peak of *Casr^Nuf/Nuf^* *α*-cells [Fig. 9(a), 9(b), and 9(d)]. There were no significant differences in the action potential frequency between *Casr^+/+^* and *Casr^Nuf/Nuf^* *α*-cells, and this was not affected by varying glucose or the addition of tolbutamide [Fig. 9(e)].

## Discussion

Our studies have shown an *in vivo* role for the CaSR in glucose homeostasis and in the regulation of pancreatic islet mass and islet hormone secretion. Thus, *Nuf* mice with a gain-of-function CaSR mutation exhibited impaired glucose tolerance, which was associated with reduced pancreatic islet mass and hypoinsulinemia as well as a lack of glucose-mediated suppression of glucagon secretion. Moreover, these findings indicate that ADH-causing mutations of the CaSR, which lead to a gain-of-function (14), may perturb systemic glucose homeostasis, and this contrasts with FHH-causing loss-of-function CaSR mutations, which have been shown to not influence glucose tolerance or insulin secretion (13). Furthermore, these findings suggest that a common coding region CaSR variant (Ala986Ser), which was reported in association with raised plasma glucose concentrations in a patient-based study (12), may have altered CaSR function in tissues involved in systemic glucose regulation. However, impaired glucose tolerance or diabetes has not been reported in ADH patients to date, and detailed investigations of glucose homeostasis in humans are warranted.

Although our studies showed CaSR activation to influence plasma glucose concentrations, we did not observe any effect of extracellular glucose on the acute signaling responses of WT or mutant Nuf CaSRs *in vitro*. Our findings are consistent with results obtained by other groups (Arthur Conigrave and Donald Ward, personal communication) but contrast with a recent study, which showed that raising the glucose concentration from 3 to 5 mM increased the Ca^2+^_i_ responses of stably expressing HEK293-CaSR cells in the presence of Ca^2+^_o_ (30). This recent study, which showed glucose to act as a CaSR allosteric activator, measured Ca^2+^_i_ responses in single cells using the fluo-8 calcium binding dye (30), whereas our study measured Ca^2+^_i_ responses in populations of HEK293-CaSR cells using the fluo-4 calcium binding dye, and these methodological differences may be contributors to the contrasting observations of these two studies.

The CaSR is a therapeutic target for calcitropic diseases (14, 38), and our studies involving the administration of ronacaleret, which is a calcilytic compound, to *Nuf* mice showed that pharmacological modulation of the CaSR may also alter plasma glucose concentrations. Ronacaleret treatment rectified the hypocalcemia of heterozygous-affected (*Casr^Nuf/+^*) mice, and this was associated with an increase in plasma insulin concentrations. Thus, these findings suggest that ronacaleret rectified the impaired glucose tolerance and hypoinsulinemia of *Casr^Nuf/+^* mice by modulating their plasma calcium concentrations, and this is in keeping with our analysis of isolated *Nuf* mice islets, which demonstrated that Ca^2+^_o_ is required for insulin release, and is also supported by a study showing that patients with chronic hypocalcemia have reduced glucose-stimulated insulin secretion (39). However, ronacaleret treatment also improved the glucose tolerance of homozygous-affected (*Casr^Nuf/Nuf^*) mice without fully normalizing their plasma calcium concentrations or altering plasma insulin or glucagon concentrations. Thus, these studies involving *Casr^Nuf/Nuf^* mice suggest that ronacaleret likely had additional effects on the glucose tolerance of *Nuf* mice, independently of altering plasma concentrations of calcium, insulin, and glucagon. The CaSR is expressed in peripheral tissues such as skeletal muscle and adipose tissue (40, 41), and it remains to be established whether ronacaleret treatment may potentially have sensitized these tissues to the actions of insulin, thereby improving glucose tolerance.

Histological analysis revealed *Nuf* mice to have a significant reduction in mean islet area, and these findings may have contributed to their reduced plasma insulin concentrations and impaired glucose tolerance. Indeed, a decrease in pancreatic *β*-cell mass is considered to be important in the pathogenesis of type 2 diabetes, as highlighted by a study of a mouse model with restricted *β*-cell expansion, which showed that a 30% reduction in *β*-cell mass is sufficient to result in impaired glucose tolerance (42). Our histological analyses also revealed individual *Casr^Nuf/Nuf^* islets to have a significant reduction in the proportion of *β*-cells compared with *Casr^+/+^* islets. Thus, these findings indicate that the CaSR may influence pancreatic islet size and the cellular composition of individual islets and suggest a role for this GPCR in the development and/or maintenance of *β*-cell mass. In support of this, mouse model studies of the *α*-2A adrenergic receptor, which is highly expressed in *β*-cells, have shown GPCR signaling to play a critical role in modulating pancreatic islet mass by inhibiting *β*-cell proliferation during the perinatal period (42). In keeping with this observation, CaSR activation was also associated with significantly reduced *β*-cell proliferation in adult *Casr^Nuf/Nuf^* islets, which may have contributed to the reduced size of *Nuf* mouse islets. However, *β*-cell proliferation was measured using the Ki67 marker, which shows proliferation over a limited timeframe, and long-term continuous labeling with the thymidine analog 5-bromo-2-deoxyuridine is required to provide a more accurate assessment of proliferation (43). Moreover, genes reported to be involved in the regulation of islet cell proliferation did not show altered expression in *Casr^Nuf/Nuf^* islets. Thus, it is possible that the gain-of-function CaSR mutation harbored by *Nuf* mice may have exerted a greater influence on islet size during the perinatal and early postnatal periods, when the *β*-cell population is undergoing a rapid expansion, and at this key developmental stage, alterations in cellular proliferation can have a substantial impact on adult *β*-cell mass and insulin secretory capacity (42). Furthermore, the CaSR may have influenced *β*-cell apoptosis, which has been shown to contribute to the reduced islet mass in humans with type 2 diabetes (44).

Isolated *Nuf* mouse islets were shown to have alterations in *β*-cell electrical activity, and *Casr^Nuf/Nuf^* *β*-cells were significantly depolarized and electrically active at low glucose concentrations. These findings suggest that the CaSR may influence the basal electrical activity of the *β*-cell, most likely by increasing background conductance (Fig. 8). In support of this, lowering the concentration of Ca^2+^_o_, which represents the major physiological ligand of the CaSR (14), rectified the increased basal activity of *Casr^Nuf/Nuf^* *β*-cells. Although the K_ATP_ channel plays an essential role in regulating the *β*-cell resting membrane potential (45), K_ATP_ channel conductance was not altered in *Casr^Nuf/Nuf^* *β*-cells, and the higher background conductance was resistant to the effects of tolbutamide. Thus, the basal hyperactivity of *Casr^Nuf/Nuf^* *β*-cells may have been mediated by a K_ATP_ channel–independent mechanism. A previous study has demonstrated that the transient receptor potential (TRP) M4 and TRPM5 ion channels regulate *β*-cell membrane potential, and activation of these channels leads to increased *β*-cell electrical activity (25). As TRPM4 and TRPM5 channels have been shown to be activated by G_q/11_-mediated phosphoinositide signaling (25), it is possible that CaSR activation induced depolarization and hyperactivity of *β*-cells by enhancing the opening of these channels. However, due to a lack of selective channel blockers, it remains to be established whether CaSR may act via TRPM4 and TRPM5 in *β*-cells. Interestingly, the increased electrical activity of *Casr^Nuf/Nuf^* *β*-cells at 1 mM glucose was not associated with an increase in basal insulin secretion. The release of insulin has been shown to be mediated by a combination of triggering effects (mediated by K_ATP_ channel closure and initiation of action potential firing) and late amplifying effects (exerted at the level of insulin granule exocytosis) (1). Thus, although *Casr^Nuf/Nuf^* *β*-cells generated action potentials at low glucose, this may not necessarily have stimulated insulin secretion. Moreover, the CaSR did not influence the overall responses of *β*-cells to stimulatory glucose concentrations; however, a reduced spike height of the glucose-induced action potentials in *Casr^Nuf/Nuf^* *β*-cells was observed. The generation of action potentials in *β*-cells is mediated by Ca^2+^ influx through the L-type voltage-dependent Ca^2+^ channel (VDCC) (46), and our observation of altered action potential height provides support for an interaction between the CaSR and L-type VDCC, as has been previously reported (47).

A key finding in this study was the presence of dysregulated glucagon secretion and altered *α*-cell function in *Casr^Nuf/Nuf^* mice. Glucagon plays a central role in systemic glucose homeostasis by stimulating hepatic glucose production, and oversecretion of glucagon contributes to the hyperglycemia in type 2 diabetes (48). The release of glucagon from *α*-cells is physiologically inhibited by elevations in glucose concentrations (26, 48). However, high glucose concentrations failed to suppress glucagon secretion from *Casr^Nuf/Nuf^* islets. We investigated whether alterations in the electrical activity of *Casr^Nuf/Nuf^* *α*-cells may have impaired the suppression of glucagon secretion following exposure to high glucose. In WT *α*-cells, glucose regulates glucagon secretion via closure of the K_ATP_ channel, and the resulting membrane depolarization leads to reduced activation of P/Q-type VDCCs that mediate the Ca^2+^ entry responsible for hypoglycemia-induced glucagon secretion (26, 37, 48). However, in *Casr^Nuf/Nuf^* *α*-cells, glucose did not induce membrane depolarization, and tolbutamide only caused a modest depolarization (approximately 2 mV), whereas this K_ATP_ channel blocker increased membrane potential by approximately 10 mV in *Casr^+/+^* *α*-cells (Fig. 9). Together, these data suggest that CaSR activation may have attenuated *α*-cell basal K_ATP_ channel activity, which impaired the membrane depolarizing effect of glucose and tolbutamide. Furthermore, *Casr^Nuf/Nuf^* mice exhibited an increase in *α*-cell numbers within individual islets, enhanced *α*-cell proliferation rates, and significantly elevated islet glucagon content. These findings highlight a potential and unique role for the CaSR in promoting *α*-cell neogenesis, but it is also possible that the hypoinsulinemia of *Nuf* mice may have led to an expansion of *α*-cells, as has previously been reported in mice with streptozotocin-induced insulin deficiency (49).

In conclusion, we have demonstrated that *Nuf* mice with a germline gain-of-function CaSR mutation have impaired glucose tolerance, which can be ameliorated by calcilytic treatment. Moreover, our findings reveal a role for the CaSR in the regulation of pancreatic islet mass and *α*- and *β*-cell function.

## Appendix

**Appendix.** **Antibody Table**

Peptide/Protein Target	Name of Antibody	Manufacturer, Catalog No., and/or Name of Individual Providing the Antibody	Species Raised in; Monoclonal or Polyclonal	Dilution Used	RRID
Primary antibodies					
Insulin	Anti-insulin	Abcam, ab7842	Guinea pig; polyclonal	0.005	AB_306130
Glucagon	Anti-glucagon	Abcam, ab92517	Rabbit; monoclonal	0.005	AB_10561971
Ki67	Anti-Ki67	Abcam, ab15580	Rabbit; polyclonal	0.002	AB_443209
Secondary antibodies					
Anti-guinea pig IgG (H+L)	Cy™2 AffiniPure Donkey Anti-Guinea Pig IgG (H+L)	Jackson, 706-225-148	Donkey; polyclonal	0.01	AB_2340467
Anti-rabbit IgG (H+L)	Cy™3 AffiniPure Donkey Anti-Rabbit IgG (H+L)	Jackson, 711-165-152	Donkey; polyclonal	0.002	AB_2307443

Abbreviations: H+L, heavy and light chains; IgG, immunoglobulin G.
