# Supplementary material for: Mutant Mice With Calcium-Sensing Receptor Activation Have Hyperglycemia That Is Rectified by Calcilytic Therapy
Source: Endocrinology. 2017 Jun 2;158(8):2486–502. doi: 10.1210/en.2017-00111 (PMC5551547; doi:10.1210/en.2017-00111)
Supplement: Supplementary file 1 [file en.2017-00111.sm1.pdf]

Supplemental Methods

Assays to assess glucose as an allosteric modulator were performed in HEK293 cells that stably expressed either the WT or mutant Gln723 CaSR proteins. These cells were generated using HEK293 T-Rex-Flp-in stable cell-lines (Life Technologies), as reported (1). Ca^{2+}_{o}-induced Ca^{2+}_{i} responses were measured using Fluo-4 Ca^{2+} assays adapted from methods previously published (2). Cells were plated in poly-L-lysine treated black-walled 96-well plates (Corning), and 12 hours later incubated in media containing 1µg/ml tetracycline (Invitrogen) to induce CaSR protein expression. On the following day, cells were incubated for 30 min in an extracellular solution composed of 140 mM NaCl, 5 mM KCl, 1 mM $MgCl_2$, 1.3 mM $CaCl_2$ and 10 mM HEPES (pH7.3), and either 3 mM, 5 mM, or 25 mM glucose (all obtained from Sigma) (3). Solutions were normalized for osmolarity (by adjusting NaCl concentrations) and pH. For studies involving ronacaleret, cells were incubated in an extracellular solution containing 3 mM glucose and 40 nM ronacaleret at 37°C. Cells were then loaded with the Fluo-4 Ca^{2+}-binding dye, which was prepared according to manufacturer's instructions (Invitrogen) and incubated for a further 30 min at 37°C (2). The Ca^{2+}_{i} assays were performed on a PHERAstar instrument (BMG Labtech) at 37°C with an excitation filter of 485nm and an emission filter of 520nm. Baseline measurements were made and increasing doses of $CaCl_2$ (0-15 mM) injected automatically into each well. The peak mean fluorescence ratio of the transient response after each individual stimulus was measured using MARS data analysis software (BMG Labtech), and expressed as a normalized response. Nonlinear regression of concentration-response curves was performed with GraphPad Prism using the normalized response at each $[Ca^{2+}]_e$ for each separate experiment for the determination of the EC_{50} (i.e. $[Ca^{2+}]_e$ required for 50% of the maximal response). Assays were performed in 8 biological replicates for each of the expression constructs. Statistical analysis was performed using the *F*-test (4).

References

1. Babinsky VN, Hannan FM, Gorvin CM, Howles SA, Nesbit MA, Rust N, Hanyaloglu AC, Hu J, Spiegel AM, and Thakker RV. Allosteric Modulation of the Calcium-sensing Receptor Rectifies Signaling Abnormalities Associated with G-protein alpha-11 Mutations Causing Hypercalcemic and Hypocalcemic Disorders. *The Journal of biological chemistry.* 2016;291(20):10876-85.
2. Leach K, Gregory KJ, Kufareva I, Khajehali E, Cook AE, Abagyan R, Conigrave AD, Sexton PM, and Christopoulos A. Towards a structural understanding of allosteric drugs at the human calcium-sensing receptor. *Cell research.* 2016;26(5):574-92.
3. Medina J, Nakagawa Y, Nagasawa M, Fernandez A, Sakaguchi K, Kitaguchi T, and Kojima I. Positive Allosteric Modulation of the Calcium-sensing Receptor by Physiological Concentrations of Glucose. *The Journal of biological chemistry.* 2016;291(44):23126-35.
4. Gorvin CM, Hannan FM, Howles SA, Babinsky VN, Piret SE, Rogers A, Freidin AJ, Stewart M, Paudyal A, Hough TA, et al. Galpha11 mutation in mice causes hypocalcemia rectifiable by calcilytic therapy. *JCI insight.* 2017;2(3):e91103.

Supplementary Figure 1

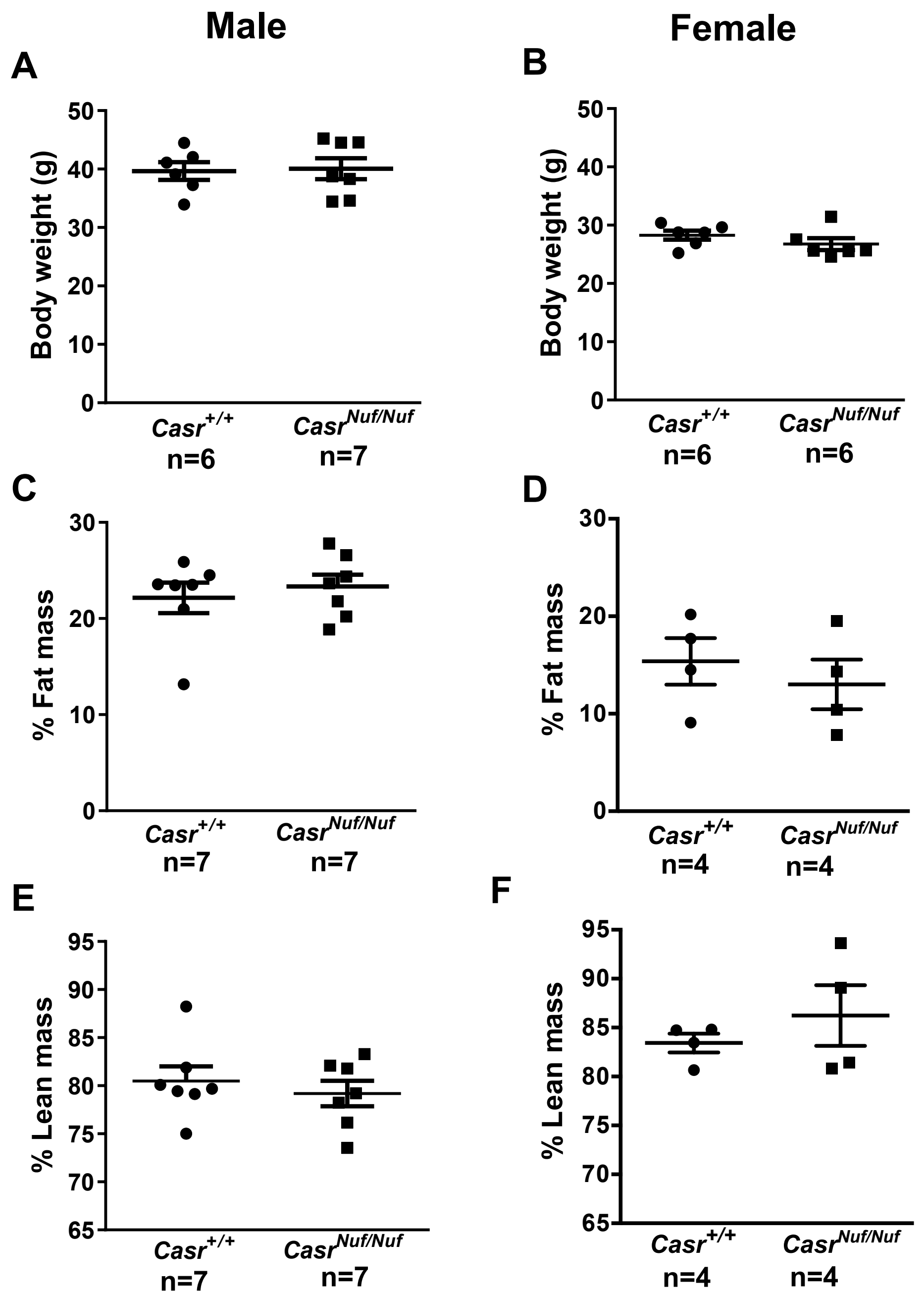

Supplementary Figure 1. Body weight and composition of *Nuf* mice. (A-B) Body weight of male and female *Casr*$^{+/+}$ and *Casr*$^{Nuf/Nuf}$ mice. (C-D) Percentage fat mass of male and female *Casr*$^{+/+}$ and *Casr*$^{Nuf/Nuf}$ mice. (E-F) Percentage lean mass of male and female *Casr*$^{+/+}$ and *Casr*$^{Nuf/Nuf}$ mice. Results are expressed as mean ± SEM.

Supplementary Figure 2

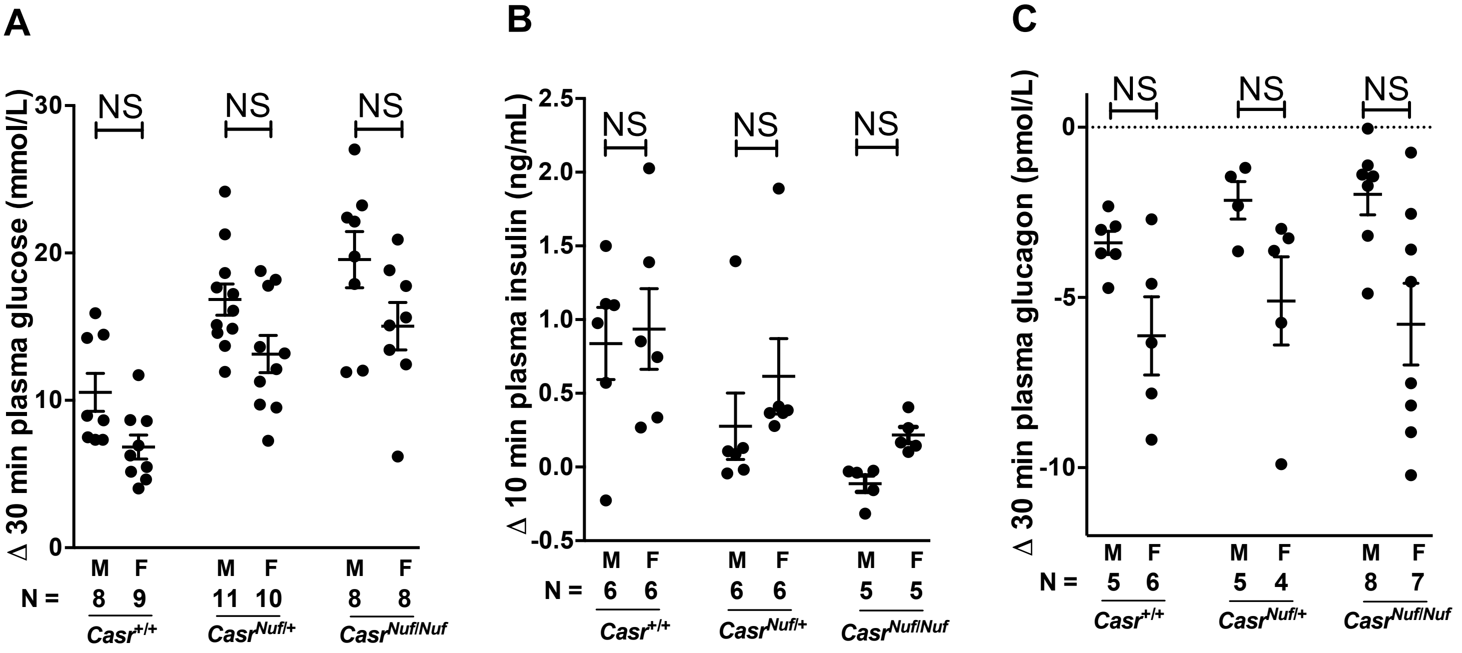

Supplementary Figure 2. Comparison of male and female responses during intraperitoneal glucose tolerance testing (IPGTT). Maximal changes (Δ) in plasma concentrations of (A) glucose, (B) insulin and (C) glucagon concentrations in male and female *Casr*$^{+/+}$, *Casr*$^{Nuf/+}$ *and Casr*$^{Nuf/Nuf}$ mice during IPGTT. Mean ± SEM values are represented by solid bars. NS, non-significant.

Supplementary Figure 3

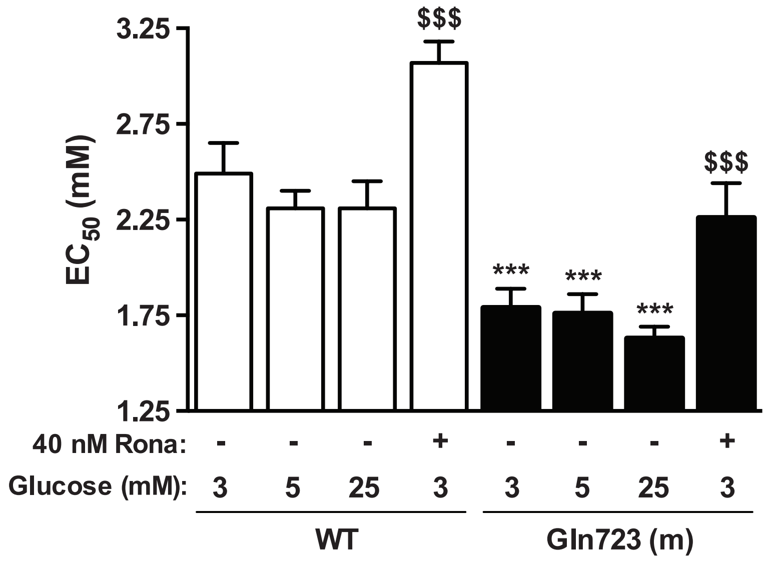

Supplementary Figure 3. Effect of glucose on the intracellular calcium EC_{50} values of the WT and Gln723 mutant CaSRs. HEK293 cells stably expressing the Gln723 (*Nuf*) mutant CaSR (black bars) showed significantly reduced EC_{50} values compared to HEK293 cells stably expressing WT CaSR (open bars, ***p<0.001), consistent with a gain-of-function, as previously reported (15,16). Increases in concentrations of glucose from 3-25 mM had no effect on the EC_{50} responses of WT or Gln723 mutant CaSR expressing cells. However, both WT and Gln723 mutant CaSR expressing cells responded to treatment with 40 nM ronacaleret (Rona) and showed significantly increased EC_{50} values compared to respective untreated cells ($$$p<0.001). Data is shown as mean ± 95% confidence intervals of 8 independent transfections. These results indicate that glucose is not an allosteric activator of the CaSR.

Supplementary Figure 4

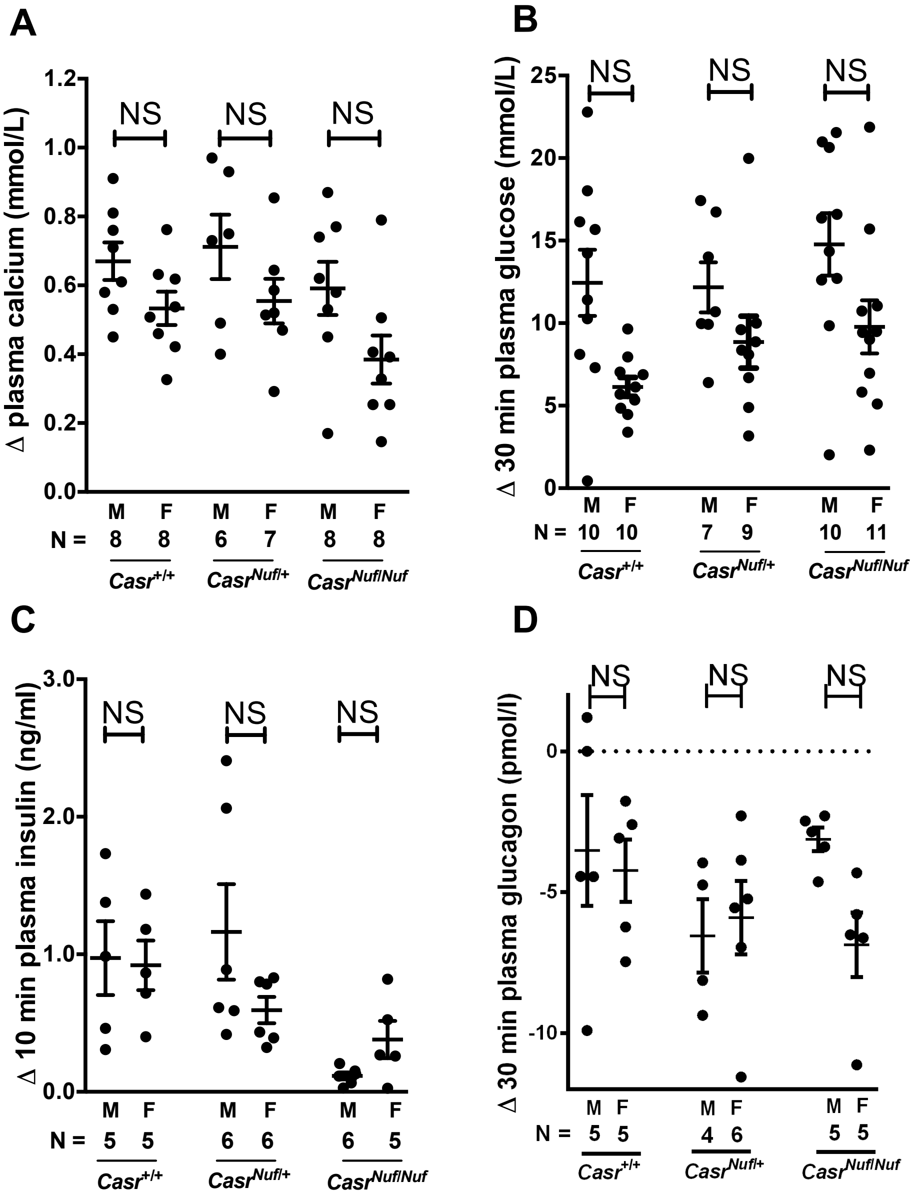

Supplementary Figure 4. Comparison of biochemical responses of ronacaleret-treated male and female mice. (A) Maximal changes (Δ) in plasma adjusted-calcium concentrations of ronacaleret-treated male and female *$Casr^{+/+}$, $Casr^{Nuf/+}$ and $Casr^{Nuf/Nuf}$* mice. Maximal changes (Δ) in plasma concentrations of (B) glucose, (C) insulin and (d) glucagon concentrations of ronacaleret-treated male and female *$Casr^{+/+}$, $Casr^{Nuf/+}$* and *$Casr^{Nuf/Nuf}$* mice during the IPGTT. Mean ± SEM values are represented by solid bars. NS, non-significant.

Supplementary Figure 5

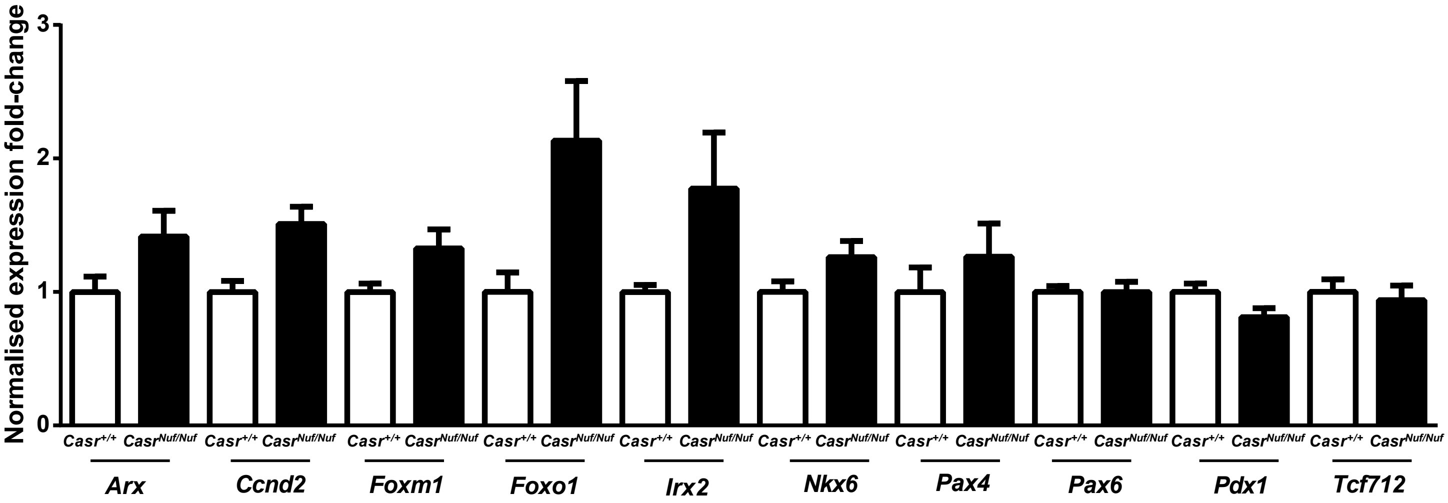

Supplementary Figure 5. Quantitative RT-PCR of genes involved in islet cell proliferation using RNA from *Casr*$^{+/+}$ and *Casr*$^{Nuf/Nuf}$ islets. The expression of genes in *Casr*$^{Nuf/Nuf}$ islet RNA reported to mediate β-cell and/or α-cell proliferation (*Arx, Ccnd2, Foxm1, Foxo1, Irx2, Nkx6, Pax4, Pax6, Pdx1, Tcf7l2*) were normalised to the mean expression of three housekeeping genes (*Actb, Eef1b2, and Gapdh*), and expressed as a fold-change of the corresponding gene expression in *Casr*$^{+/+}$ islet RNA. Results from n=5 male and n=6 female mice have been combined (n=2 technical replicates per mouse) and are shown as mean ± SEM. Alterations in gene expression between *Casr*$^{Nuf/Nuf}$ and *Casr*$^{+/+}$ islets were assessed using the Kruskal-Wallis test for multiple comparisons.
